# Metabolic dysfunction-associated steatotic liver disease prevalence and risk factors in inﬂammatory bowel disease in tertiary center

**DOI:** 10.1590/1806-9282.20231321

**Published:** 2024-07-19

**Authors:** Lívia dos Remédios Pamplona de Oliveira, Tarsila Campanha da Rocha Ribeiro, Carlos Alberto Mourao, Maria Antônia de Lima Barra, Mariana Hippert Gonçalves Silva, Luis Pordeus Shafee, Sarah Mendes Zacarias, Lenilton da Costa Campos, Helena Maria Giordano Valério, Júlio Maria Fonseca Chebli

**Affiliations:** 1 Universidade Federal de Juiz de Fora, Hospital Universitário – Juiz de Fora (MG), Brazil.; 2 Universidade Federal de Juiz de Fora, Department of Physiology – Juiz de Fora (MG), Brazil.

**Keywords:** Inflammatory bowel diseases, Crohn's disease, Ulcerative colitis, Nonalcoholic fatty liver disease, Metabolic syndrome, Liver fibrosis

## Abstract

**OBJECTIVE:**

The aim of this study was to evaluate the prevalence and risk factors related to metabolic dysfunction-associated steatotic liver disease in inflammatory bowel disease patients.

**METHODS:**

This is a cross-sectional study conducted on adults with inflammatory bowel disease from 2019 to 2021. Metabolic dysfunction-associated steatotic liver disease encompasses patients with steatosis and at least one cardiometabolic risk factor. Patients with alcohol consumption ≥20 g/day, chronic liver diseases, or methotrexate use were excluded.

**RESULTS:**

Almost 140 patients were included: 67.1% were female, with a mean age of 49.7±13.7 years, and 63.6% had Crohn's disease. The mean duration of inflammatory bowel disease was 9.7±7.9 years. Metabolic dysfunction-associated steatotic liver disease was observed in 44.3% and advanced liver fibrosis was excluded in 63.5% by Fibrosis-4. Patients with metabolic dysfunction-associated steatotic liver disease were older (p = 0.003) and had a higher number of metabolic syndrome components (2.9±1.1 versus 1.6±1.0; p<0.001), greater abdominal circumference (p<0.001), and body mass index (p<0.001). The only factor related to inflammatory bowel disease associated with metabolic dysfunction-associated steatotic liver disease was disease duration (11.6±9.5 versus 8.3±6.2; p = 0.017). A higher number of metabolic syndrome components and obesity increase by 2.2 times and an altered waist circumference by 2.6 times the occurrence of metabolic dysfunction-associated steatotic liver disease.

**CONCLUSION:**

A high prevalence of metabolic dysfunction-associated steatotic liver disease was observed in patients with inflammatory bowel disease, with the main risk factors being associated with metabolic syndrome predicting it, but not with inflammatory bowel disease features and/or its treatment.

## INTRODUCTION

Nonalcoholic fatty liver disease (NAFLD) is among the most common causes of chronic liver disease, mainly related to insulin resistance and metabolic syndrome (MS), after the exclusion of secondary causes^1^. Its spectrum ranges from simple steatosis to advanced fibrosis (AF) and hepatocellular carcinoma^2^. Recently, a new NAFLD nomenclature was proposed to contemplate its physiopathology and reduce the impact of subject stigmatization based mainly on exclusion criteria. The new term "metabolic dysfunction-associated steatotic liver disease (MASLD)" comprises hepatic steatosis (HS) associated with one out of five cardiometabolic risk criteria in individuals without significant alcohol consumption^3^.

The prevalence of MASLD is increasing worldwide, in parallel with the obesity epidemic and the expansion of cardiovascular diseases, being estimated at 25–30% of the general population^4-6^. Despite the scant data regarding the prevalence of MASLD in Brazil, it is known that in Latin America, the disease is highly prevalent, occurring in 24% of the population^7^. Recent studies indicate that the prevalence of NAFLD in IBD patients varies from 1.5 to 55%^8,9^. Both diseases are multifactorial, involving environmental, genetic, and immunological determinants^10^.

In most cases, MASLD is linked to insulin resistance and is considered the hepatic manifestation of MS^2^. However, the pathogenesis of MASLD in the IBD population may involve specific risk factors, such as chronic inflammatory response, drug hepatotoxicity, frequent steroid use, malnutrition, previous intestinal resection, and intestinal dysbiosis^8^. It is postulated that MASLD in IBD patients may occur through two distinct phenotypes, one triggered by factors directly related to IBD and the other associated with the components of MS^10,11^.

Earlier studies have found an association between MASLD and features of IBD, such as disease severity and duration, previous intestinal resection, and drugs used in the treatment^10,11^. Conversely, anti-tumor necrosis factor (anti-TNF) agents may have a protective role against the occurrence of MASLD^12,13^. However, more recent studies have pointed out that components of MS are more critical to the occurrence of MASLD in IBD.

With advancing knowledge of IBD and its therapeutic arsenal, the disease phenotype has changed in recent years, with steatosis and liver disease increasingly reported in patients with IBD. However, data regarding predisposing factors for the occurrence of steatosis are still conflicting. With the implementation of new nomenclature and the absence of studies that evaluate the prevalence of MASLD and its risk factors in our country, the present study was proposed.

## METHODS

This cross-sectional study included patients over 18 years old followed up at the IBD Reference Center of the University Hospital of the Federal University of Juiz de Fora (HU-UFJF) from January 2019 to December 2021. The diagnosis of IBD was established by clinical, endoscopic/histological, and/or imaging criteria. Patients with alcohol consumption >20 g/day, chronic liver disease, or use of methotrexate were excluded. This study was approved by the Human Research Ethics Committee of HU-UFJF (CAAE 06129419.0.0000.5133) and participants signed an informed consent form before inclusion.

Data on clinical–demographic and IBD features (disease type and location, age at diagnosis and disease length, current and previous treatment) were collected. Disease activity was defined by a colonoscopy showing the presence of ulcers in CD or a Mayo score ≥2 in UC^14^ and/or compatible imaging and/or biochemical findings (C-reactive protein>6 mg/dL).

The diagnosis of metabolic syndrome was established according to the National Cholesterol Education Program's Adult Treatment Panel III (NCEP-ATP III) criteria: elevated waist circumference (≥94 cm in males or ≥80 cm in females); triglycerides ≥150 mg/dL; HDL cholesterol <40 mg/dL in males or <50 mg/dL in females; elevated blood pressure (systolic ≥130 and/or diastolic ≥85 mm Hg); or fasting glucose ≥100 mg/dL^15^. Biochemical assessment included metabolic, liver, and inflammatory profiles, as well as viral and autoimmune markers.

The HS diagnosis was established using an imaging technique (ultrasonography, tomography, or magnetic resonance) at the time of inclusion or in the previous 6 months, if available, and carried out in our service. The diagnosis of MASLD was defined by steatosis and at least one cardiovascular risk factor, as recently proposed by the American and European Association of Liver Diseases.³

For assessing advanced liver fibrosis, the Index for Liver Fibrosis-4 (FIB-4) was used as a noninvasive test, widely validated, and recommended for screening in a low prevalence population of liver fibrosis. Values lower than 1.3 or higher than 2.67 excluded or confirmed the diagnosis of AF. Intermediate values were considered indeterminate and nondiagnostic^16,17^.

Continuous variables were described as mean and standard deviation and categorical variables were described as frequency and percentage. Comparison between continuous variables was established using the Student's t-test, while the chi-square test or Fisher's exact test was used to evaluate categorical variables. All tests were two-tailed and adopted a significance level of 5%. For MASLD prediction models, binary logistic regression models were performed. The choice of independent variables was based on previous univariate analyses and clinical criteria previously established in the literature. The goodness of fit of the regression model was verified by the Omnibus test and the respective ROC area under the curve (AUC). Inferential and modeling analyses were carried out using the Jamovi version 2.3 application.

## RESULTS

A total of 217 patients were evaluated, of which 77 were excluded (63 lost to follow-up, 5 had alcohol use disorder, 5 had chronic liver disease, and 4 were using methotrexate). Of the 140 patients included, 67.1% were female, with a mean age of 49.7±13.7 years, and the majority had Crohn's disease (63.6%). HS was evident in 45% of the sample. The diagnosis of MASLD was established in 44.3%. Only 20% of patients had elevated alanine aminotransferase, while advanced liver fibrosis was present in 6.5% of cases and could be excluded using the noninvasive FIB-4 score in 63.5% of patients. The clinical–demographic characteristics of the studied population are described in Table 1.

**Table 1:** Clinical–demographic characteristics of inflammatory bowel disease patients with steatotic liver disease associated with metabolic dysfunction.

		Total (n = 140)	Without MASLD (n = 78)	MASLD (n = 62)	p-value
Women % (n)		67.1 (94)	52.1 (49)	47.9 (45)	0.222
Age (years)		49.7 ± 13.7	46.6 ± 14.3	53.5 ± 11.9	0.003
Age at diagnosis (years)		39.9 ± 13.1	38.3 ± 13.5	41.9 ± 12.3	0.102
Illness duration (years)		9.7 ± 7.9	8.3 ± 6.2	11.6± 9.5	0.017
Type of IBD					
	CD/UC % (n)	63.6 (89)/36.4 (51)	55.1 (49)/56.9 (29)	44.9 (49)/43.1 (22)	0.836
Location of CD					
	L1/L2/L3/L4 % (n)	31.5 (28)/20.2 (18)/46.1 (41)/2.2 (2)	30.6 (15)/14.3 (7)/55.1 (27) -	32.5 (13)/27.5 (11)/35 (14)/5 (2)	0.091
CD phenotype					
	B1/B2/B3 % (n)	31.5 (28)/36 (32)/32.6 (29)	30.6 (15)/38.8 (19)/30.6 (15)	32.5 (13)/32.5 (13)/35 (14)	0.862
	Perianal disease % (n)	18.1 (21)	57.1 (12)	42.9 (9)	0.708
Location of UC					
	E1/E2/E3 % (n)	9.8 (5)/45.1 (23)/45.1 (23)	6.9 (2)/41.4 (12)/51.7 (15)	13.6 (3)/50 (11)/36.4 (8)	0.556
	Previous surgery % (n)	21 (29)	58.6 (17)	41.4 (12)	0.730
Treatment					
	Anti-TNF therapy % (n)	35.7 (50)	56 (28)	44 (22)	0.960
	Steroid use % (n)	28.6 (40)	55 (22)	45 (18)	0.914
	Active disease % (n)	60 (84)	51.2 (43)	48.8 (62)	0.187
	Number of SM components	2.1 ± 1.3	1.6 ± 1.0	2.9 ± 1.1	<0.001
	Blood glucose	100.2 ± 29.8	92.3 ± 12.4	110 ± 40.6	<0.001
	Glycated hemoglobin	5.5 ± 1.4	5.2 ± 0.5	5.9 ± 1.9	0.003
	Insulin	10 ± 5.4	8.5 ± 5.0	11.9 ± 5.4	<0.001
	HOMA-IR	2.5 ± 1.8	2.0 ±1.4	3.3 ± 2.1	<0.001
	Triglycerides	146.7 ± 82.2	131.3 ± 67.1	166.2 ± 95	0.012
	Total cholesterol	187.7 ± 42.2	185.2 ± 43	190.9 ± 41.6	0.427
	HDL	49 ±12.7	50.5 ± 13.8	47 ± 11	0.117
	LDL	109.6 ± 35.8	108.4 ± 37.7	111.1 ± 33.5	0.665
	AST	23.5 ± 9.6	23.1 ± 7.6	24.1 ±11.7	0.582
	ALT	21.3 ± 11.2	20.1 ± 11.2	22.8 ±11.2	0.168
	Albumin	4.2 ± 0.2	4.2 ± 0.2	4.3 ± 0.2	0.291
	Creatinine	0.8 ± 0.2	0.8 ± 0.2	0.7 ±0.2	0.221
	CRP	7.4 ± 14.1	7.6 ± 16.8	7.2 ± 9.3	0.921
	ESR	21.8 ± 19.8	21.7 ± 23	22.1± 15.1	0.930

MASLD: metabolic dysfunction-associated steatotic liver disease; IBD: inflammatory bowel disease; CD: Crohn's disease; UC: ulcerative colitis; L1: ileal; L2: colonic; L3: ileocolonic; L4: upper gastrointestinal tract; B1: non-stenosing, nonpenetrating; B2: stenosing; B3: penetrating; E1 proctitis; E2: left colitis; E3: pancolitis; SM: metabolic syndrome; CRP: C-reactive protein; ESR: erythrocyte sedimentation rate.

**Figure 1:**
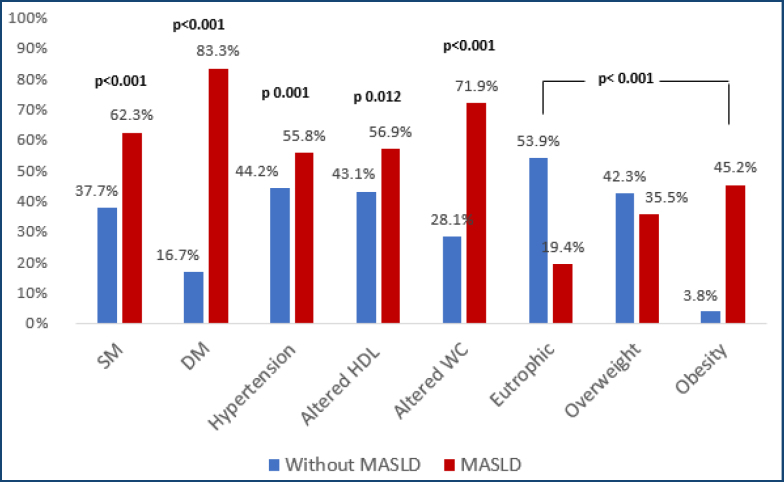
Correlation between components of the metabolic syndrome and the presence of steatotic liver disease associated with metabolic dysfunction. SM: metabolic syndrome; DM: diabetes mellitus; HDL: high-density lipoprotein; WC: waist circumference; MASLD: metabolic dysfunction-associated steatotic liver disease.

Patients with MASLD had a higher frequency of MS, diabetes, hypertension, altered waist circumference (CW), and obesity (Figure 1). Furthermore, the MASLD patients had more ATP III metabolic risk factors, were older, and had a higher length of disease than those without MASLD. Conversely, data related to IBD (type, extent, phenotype, treatment, disease activity) were unrelated to the presence of MASLD (Table 1).

Multivariate analysis showed that MASLD was independently associated with a higher number of ATP III metabolic risk factors, obesity, and an altered CW, with an AUC of 0.85 (Table 2).

**Table 2:** Predictive factors associated with the presence of steatotic liver disease in patients with inflammatory bowel disease.

Variables	OR (95%CI)	p-value
Number of SM components	2.20 (1.47–3.29)	<0.001
Presence of obesity	2.29 (1.18–4.42)	0.014
Altered WC*	2.98 (0.97–9.12)	0.056

SM: metabolic syndrome.

* Waist circumference greater than 94 cm in males and 80 cm in females.

## DISCUSSION

The present study showed a high prevalence of MASLD (44.3%) in IBD patients, with the main associated predictors related to MS risk factors. Although most individuals do not have advanced liver fibrosis, it was predicted in 6.5% of our casuistic, even though they were asymptomatic, showing the importance of screening for MASLD in this population, given its silent progressive behavior.

With the global obesity epidemic and the increasing prevalence of cardiovascular events, HS has been reported in 25% of the world's population. Greater awareness of IBD and an improved therapeutic arsenal of the disease have influenced the change in IBD patients' phenotype, with a more significant occurrence of overweight or obesity. A growing interest in the prevalence of HS in IBD patients has occurred in recent years, reported between 8.6 and 54%, which may vary according to the method used for the diagnosis^8,18^. The presence of AF or liver cirrhosis is reported in 18.3%^10,12^. The prevalence of HS in our study was similar to that reported in previous studies.

The pathophysiology of HS in IBD patients still needs to be well established. Bessissow et al. identified the activity and duration of the disease, as well as previous intestinal resection, as independent risk factors for HS^19^. Some of these results were replicated in subsequent studies that observed a correlation with corticosteroids or methotrexate use^10,20^. Conversely, therapy with anti-TNF could have a protective effect^12,20^. In our study, only the disease duration was longer in those with MASLD compared to patients without MASLD; however, it did not remain significant in the multivariate analysis. Data inherent to the type, extent, phenotype, or previous treatment, including intestinal resection, were not associated with MASLD.

More recent studies, such as the one by Palumbo et al., showed older age, higher BMI, and higher triglyceride levels as independent risk factors for HS^10^. Regarding the AF presence, Palumbo et al. found age and BMI predictors of its occurrence. In our series, older age, duration of the disease, and several MS diagnostic criteria (DM, hypertension, obesity, low HDL, and increased WC), as well as a higher number of ATP III MS components, were associated with MASLD. However, only a higher number of ATP III MS components, obesity, and an altered WC were independently associated with MASLD in the multivariate analysis. All these data are in line with more recent studies^2,9^.

Noninvasive diagnosis of liver fibrosis through tests such as FIB-4 is increasingly being recommended, especially in populations at low risk of AF, to exclude it and early referral to a specialist for those "at risk" of AF^18,21,22^. In our casuistic, we could exclude the presence of AF in 63.5% of cases and diagnose it in 6.5%, despite awareness of FIB-4 positive predictive value limitations. However, with this strategy, only 20 out of the 62 patients with MASLD would need to continue diagnostic investigation using more advanced methods. Our results agree with those of Trifan et al. that most patients undergoing a more sensitive method for AF diagnosis (liver transient elastography) did not present it^23^.

The main drawback of our study was that the methods used for HS diagnosis were heterogeneous and, in most cases, established by ultrasonography (US) findings. It is known that the diagnostic accuracy of the US may be inadequate for mild steatosis recognition, as it is also an operator-dependent technique, with MRI being the most accurate method, despite the cost that often limits its use^20^. Furthermore, as ours is a tertiary hospital with reference services in IBD and hepatology, there may have been some selection bias, with more severe patients being included in the study, which should not reflect the national scenario.

To the best of our knowledge, this is the first study that used the new nomenclature established for steatotic liver disease, which consists of better-established criteria that consider the metabolic nature of the physiopathology. The high prevalence of MASLD in patients with IBD makes it necessary to have a plan to prevent progression to more severe forms of disease. It is mandatory for the professional responsible for managing these patients to be aware of the interaction between IBD and MASLD to adopt screening measures and refer "at-risk MASLD" to a hepatologist.

## CONCLUSION

Our findings supported the reported high prevalence of MASLD in IBD patients and its close relationship with MS risk factors, highlighting the importance of careful screening and management of MASLD in this scenario.
